# Dentistry Not a Specialty in Medicine

**Published:** 1887-01

**Authors:** Norman W. Kingsley


### Independent Practitioner.

Vol. VIII. January, 1887. No. 1.

### Original Communications.

Note.—No paper published or to be published in another journal will be accepted for this department. All papers must be in the hands of the Editor before the first day of the month preceding that in which they are expected to appear. Extra copies will be furnished to each contributor of an accepted original article, and reprints, in pamphlet form, may be had at the cost of the paper, press-work and binding, if ordered when the manuscript is forwarded. The Editor and Publishers are not responsible for the opinions expressed by contributors. The journal is issued promptly, on the first day of each month.

### DENTISTRY NOT A SPECIALTY IN MEDICINE.

BY NORMAN W. KINGSLEY, D. D. S.

Annual Address before the New England Dental Society.

When I received the invitation from your committee to be present on this occasion, I said, involuntarily, from a force of habit—no. But the compliment paid me in the invitation, together with the flattering reception accorded me in Boston on a former occasion, made me hesitate in sending a negative reply.

When I have something to say a little out of the ordinary practical and scientific talk of dental societies, I like to come to Boston with it. The old fancy that wisdom was born in the East and spreads from that point of the compass, induces me to seek this locality and take advantage of the myth. As my professional audience, other than that now before me, lies to the west of us, my words may possibly gain a factitious importance by being delivered in Boston. I realize in advance that some things I am about to say will not be accepted, and are liable to be severely criticized.

I have for some years been drifting in a conflict of opinion upon a subject of universal interest to us, until a period of positive conviction has arrived, and in my present discourse I shall present my reasons for believing that dentistry is not a specialty of medicine. Such an assertion requires some boldness, in view of the prevalence of a contrary opinion, associated as it has been with the idea that, in some indefinite sort of way, to be regarded as specialists in medicine gives us character and dignity, and invests us with the right to be called scientists. This desire to be considered medical specialists has its foundation in a notion that dentistry, pure and simple, is ignoble, to practice it degrading, and that to stand well in society we must resolve that we are not dentists, but medical doctors practicing a specialty. It has also been stimulated by that spirit of contrariness which inspires children to cry for that which is denied them, and as the medical profession, as a whole, have denied that dentistry is a specialty of medicine and is only a mechanical trade, dentists have got very much in the habit of denying that dentistry is a mechanical trade, and on all occasions asserting and reasserting that they belong to the great medical fraternity. That they do sometimes prescribe medical remedies, apply leeches and cut gum-boils is true, but there is not an old housewife in the country who is not more of a medical doctor and better capable of treating the diseases of the family than the majority of dentists, even if they have the medical degree.

Asserting that we are medical practitioners does not make us so, any more than the three Tailors of Tooley Street became the "People of England" when they met in solemn conclave and passed their famous resolution to that effect. One is reminded of the old fable of the ass with a lion's skin. The skin was the skin of a lion, but the bray was that of an ass.

All this betrays upon the face of it a consciousness of demerit—a consciousness that they are claiming what they are not entitled to, on the Jew principle of asking more for a thing than it is worth and accepting what can be obtained.

The assertion of the medical fraternity that dentistry is only a mechanical trade because dentists make artificial teeth, is no wider of the mark than the claim of some dentists that, because they can stop a local ache or wrestle with pyorrhœa alveolaris, they are thus constituted specialists in medicine.

The true status of dentistry is distinctly separated from either of these incongruous claims. Now, that there may be no ambiguity of terms, let me say, before going further, that by dentistry I mean to include every branch and department known under that name, and a dentist in the full sense of the term, in the present stage of the art, is one who understands and can practice each and every specialty of it. The American Dental Association, as is well known, is divided up into seven or eight sections. I was recently called upon to declare to which section I wished to attach myself. My reply was: "To no *section;* I am not the eighth part of a dentist, to which seven other parts must be added to make a whole."

In passing, I wish to remark that that sectional plan, copied after the American Medical Association, is not suited to our condition. Whatever may be the result in the Medical Association, I am confident that all the members of our National Association would return from its meetings with more benefit if the sections were abolished.

I take pride in being a dentist in the full sense of the term. No man ever applied to me for a service that came within the range of dentistry, to whom I was obliged to say, "that is not my specialty; I shall have to send you to some one else."

A dentist may be an oral surgeon, but an oral surgeon is not a dentist. A dentist may be an excellent anatomist, physiologist, chemist, microscopist, artist or mechanic, but neither one of these practiced to perfection makes him a dentist.

I desire to make the distinction very marked. Oral surgery, which is an almost infinitesimal part of dentistry (as dentistry is practiced daily the world over), is unquestionably a specialty or department of general medicine. But dentists who affect oral surgery, or who occasionally perform some trifling operation, have not much more claim to be called surgeons than has the old farmer who opens a boil for one of his laborers to let out the core. Oral surgery, as practiced by dentists, is only a very small but not unimportant specialty of dentistry; it occupies the debatable ground between dental and general surgery, but as an essential department of practical dentistry, its importance has been magnified by those practitioners who are more skilled in surgery than they are in dentistry.

I am, therefore, prepared to affirm that dentistry is not a specialty of any other science or art, but is a profession in itself, as separate and distinct from all others as any other calling or vocation is distinct from every other.

Dentists are very much in the habit of speaking of their occupation in one and the same breath, as a "profession" and as a "specialty," but I doubt very much if many of them could give any good reason why it should be called a profession. They use the term because it sounds well, is a little higher-toned, and carries a degree of importance with it.

But dentistry is a profession. It is a profession because it is a vocation of beneficence. This is so patent that I need not attempt to prove it, or enlarge upon it. Millions are on the earth to-day who call us blessed because of the comfort we have given them and the benefit they have derived from us.

Dentistry is a profession by universal acknowledgment. All things in this world, physical, political, social or moral, by the law of equilibrium very soon find their own level. A man in his egotism, conceit and vanity, may assert this or that of himself, but whatever it may be, the great common sense of the "plain people" (as Mr. Lincoln fondly called them) puts him in his true place.

Dentistry has been an organized science for more than a generation, and has been called a "Profession" by universal consent, by the cultured and uncultured, as well as by its own practitioners. Even the highest authorities in medical literature refer to dentistry, not as the "Dental Specialty of Medicine," but as a "Profession." That most distinguished of medical authors, the late Prof. Frank H. Hamilton, in a letter to the Odontological Society of New York, written last February, said :

"To Americans, by almost universal consent, is given the chief credit of having brought dentistry from a simple mechanical art to the rank of a science, and of having established for itself a just claim to the title of a 'Learned Profession.'"

Your own beloved Holmes, distinguished alike as an author and a savant—one who does not use the English language recklessly or without meaning—sent to the same society last winter this toast :

"The Dental Profession and this Association as its worthy representative." It has established and prolonged the reign of beauty; it has added to the charms of social intercourse and lent perfection to the accents of eloquence; it has taken from old age its most unwelcome feature and lengthened enjoyable human life far beyond the limit of the years when the toothless and purblind patriarch might well exclaim, 'I have no pleasure in them.'"

This designation of it as a "profession" is not an assumption like that of the barber, the dancing master, or the itinerant phrenologist; it is entitled to this distinction because the mastery of it as a science or an art involves a considerable knowledge of many other sciences. Its resources are not only nearly all the sciences, but, in an equal degree, nearly all the arts. Hardly an art, from plumbing to sculpture, but has its prototype in some branch of dentistry, and yet it is not a department or specialty of any one of them.

Suppose that in some kind of manufacture in which I might be engaged I was using strips of iron punched full of holes, and wanted a machine to make them. Such a machine could be made by a combination of well-known movements; for example, a treadle and driving wheel borrowed from a foot lathe; the movement of the punch from a sewing machine, and the feeding apparatus from a saw-mill. Such a machine would be distinctly a new machine, and patentable as such, although deriving all its principles from old sources. It would not be an improvement on a foot lathe, nor on a sewing machine, nor again on a saw-mill. The new combination and new application of old principles would make it an entirely new machine. It is so with dentistry.

While many of its processes are mainly of a mechanical nature, it is not a mechanical trade, inasmuch as a mechanical trade is governed by fixed rules and a routine of labor, in which each workman is a servile imitator of the pattern given him, and can become master of his trade without any knowledge beyond its details. His brain is not constantly called upon to apply established principles to entirely new conditions and surrounding circumstances. The distinction which I would make between a trade and a profession is that, while the latter may employ the identical methods of the former, the judgment and the inventive faculties of the practitioner must be in active exercise to apply those principles and those methods to constantly varying conditions.

The predominating feature and characteristic of dentistry, that which removes it farther than all else combined from medicine, is the mechanical character of its methods. We might as well try to blot out the inevitable laws of this universe as to ignore this great fact: dentistry, as a profession, has for its corner-stone and its entire foundation mechanics applied by a knowledge of the various sciences. So much alike are the methods of the gold and silver jeweler to dentistry, that the acquirement of one would be a partial education for the other; yet making gold and silver jewelry is not a profession; it is a trade. Dentistry, while using the same mechanical processes, is obliged to add invention in their application to every case.

The methods of the painter and sculptor are the methods of the mechanic. But portrait, figure and landscape painting and sculpture are branches of fine art, and the vocation is a profession, not a trade. Figure painting and sculpture, particularly, require a better knowledge of general anatomy than dentistry does; but who ever heard a sculptor assert that he was for that reason "a specialist in medicine?" Michael Angelo's distinction and pre-eminence as the master artist of the world was due to his anatomical knowledge; and Sir Charles Bell made a science for artists in his "Anatomy and Physiology of Expression."

Blot mechanics from dentistry, and you might as well blot the sun from our terrestrial universe; chaos irredeemably follows.

A few dilettanti may confine themselves to operations on the natural teeth, and scout with indignation the idea that they are mechanics, but every step of the operation in filling a tooth is purely mechanical. It requires nice skill to be sure, but the skill is mechanical. That which dignifies it, bringing it above ordinary mechanics, is the fact that it is performed upon living organisms, and that which makes the operation professional is the knowledge of anatomy, pathology, etc., which discriminates in directing the mechanical treatment.

We must form our judgment of dentistry as it is to-day, in the year of grace 1886, and not as it may be in some utopian future, when the race shall have become so far advanced in the knowledge and application of hygienic principles, and all transmitted tendencies to deterioration have been stamped out, that teeth no longer need repair. Statistics of dentistry throughout the world to day would undoubtedly show that three-fourths of the combined aggregate income of the profession is derived from the exercise of mechanical skill, pure and simple, and that I believe without counting operative dentistry as a mechanical performance.

Dentistry is not a specialty of medicine, because its chief and predominating characteristics are utterly unlike anything which is taught in medicine, requiring for their successful performance natural faculties and acquirements that are entirely distinct from the practice of medicine.

Dentistry may be said to be more nearly allied to medicine than to any other vocation, but an analysis may even question that. Laying aside, for the sake of the argument, what we consider as the unprofessional character of exhibitions of dental workmanship, and also the fact that such work is prosthetic in its intent, would we not be quite justified in making contributions to an industrial exhibition which was confined to works of art, including all objects of art in gold, silver and porcelain? Where are there any finer specimens of the art of gold working than some of the so-called bridge-work of recent times?

In passing, I cannot refrain from paying a tribute to such work shown by Dr. J. Rollo Knapp, of New Orleans, at the last meeting of the American Dental Association. They were brilliant mechanical achievements, and ought to make men, who have been trying to cut off mechanics from dentistry, hide their heads with shame. Men who can fill a tooth and nothing more, who could not execute such a piece of prosthetic dentistry to save their lives, assume an air of superiority and prate about relegating the mechanics of dentistry to the shop and to mere mechanics. Wipe out of dentistry everything belonging to mechanics, and you will have taken away all the brains, and cut the head off close to the tail.

If all the workers in metals, gold, silver, brass, iron or steel—if all the workers in wood, carvers, cabinetmakers and builders—if all the workers in pottery, moulders, porcelain-makers, and decorators, together with all the artists, painters and sculptors, were suddenly and simultaneously destroyed by some strange cataclysm or epidemic, those arts would not be lost; for in the ranks of the dentists could be found skilled experts in every one of them, and this comprehensive combination of natural faculties and acquirements is not to count against them, for if in the same grand catastrophe all the scientists of certain classes were carried off, the same sciences could be fully taught by dentists.

In the daily practice of dentistry can be found anatomists, physiologists, pathologists, histologists, biologists, microscopists, chemists, botanists, geologists and metallurgists.

Where in all the wide range of human employments is there another vocation, no matter whether you call it a profession or a trade, of which such a statement can be made?

That which makes dentistry as a science kindred to medicine as a science is the fact that it deals with a small but important part of the human economy. But the equally great fact that its methods are entirely distinct, requiring special education and special training, make it an independent science, and in no sense subordinate to the other.

The training of a dental student for his professional career is totally unlike that required by a medical student. Medicine involves hospitals and bedside practice, but dentistry involves, along with a study of the sciences, training the fingers, first, second, and all the time.

If dentistry be a specialty of medicine, where was the necessity for State laws regulating its practice, separate from those already in existence for the regulation of medicine? Does not the greater necessarily include the less? Does general surgery, which is a department of medicine, or oral surgery, which is a specialty, need special laws? Are not the laws of all the States, passed in the interest of medicine, quite sufficient to protect oral surgeons as well as oculists, gynecologists, and what not? Nevertheless, the State of New York, which is behind no other civilized community, did not include dentists among its exempts from jury duty, while it had exempted physicians, even from the organization of the government. I claim for myself the honor of obtaining from our Legislature a special law which exempted dentists equally with physicians from that annoying service.

Dentistry became an independent profession, not through any spirit of rebellion against the medical profession, but from sheer necessity. The fathers of dentistry in this country were graduates of medicine, and hoped to dignify their vocation by grafting it upon medicine, and have the theory and practice taught in medical schools. Their application was refused, and the history of dentistry as an independent, progressive and scientific organization began, and to-day the wondrous fact is the astonishment and admiration of the scientific world. We have more than a dozen independent institutions of learning which teach everything that a dentist needs to know. We have an independent literature which is not indebted to medicine so much as it is to other sciences.

Anatomy, physiology, histology, microscopy, chemistry, etc., are not *medical* studies. They are *sciences* upon which medical and other studies are based.

We have an independent journalism, larger than the total of medical journalism when our history began. We have independent national, state and local organizations that are vital, active and progressive, and what might once have been, viz., dentistry taught and practiced as a specialty of medicine, cannot now, in the very nature of things, ever be brought about. Even if it were possible at this day to blot out all the organizations—literary, social, educational and scientific—which now mark its independence, and reduce the profession to a mere section of medicine, no man with any pride in his calling or desire for its highest attainments would consent to it. The attempt to make it so now emasculates and degrades it. Furthermore, the tendency of the times is against it. Great social and political problems are being rapidly worked out. The growth of States and of capital is toward centralization. The growth of science is by segregation; the cellular theory is beautifully exemplified in its development.

As knowledge increases, the sciences divide and subdivide into specialties, and the specialty, through its independent and frequently original methods of investigation, speedily takes rank as a distinct science. In separate organizations the sciences will continue to advance; centralize them and make them specialties of one another, and the structure becomes top-heavy and crumbles.

Naturally, my audience will turn to the problem of the best method of preparing men for the practice of this independent profession. The day was in the memory of some of my hearers when the ideal philosopher had acquired and possessed the sum of human knowledge. It is not a century since a cyclopædia of modest dimensions would contain all that the human mind had gathered of all the sciences, in all the ages, which was worth knowing and preserving. The day is when dentistry—once an empirical trade, and now an independent profession—requires from those who would stand in the front a devotion to study and an active acquaintance with the current advance of allied sciences, greater than was demanded of the philosopher of a former age, who held within his own brain the entire sum of human knowledge.

We must not forget that it is these times and this generation which demands our notice. We are not making plans for the millennium, nor for some ideal and Arcadian state of existence. The duties of to-day crowd upon us, and to meet fully the to-day is the very best way to be prepared for the morrow. The morrow grows out of and upon the to-day. To plan for to-morrow and leave to-day is lunacy; to meet fully the emergency of to-day, even giving no thought to the morrow, is the foundation of wisdom. Of course, I do not use the terms "to-day" and "to-morrow" in their literal sense, but make them figures of speech to stand for the present time and the great unknown future.

The fiat of nature that man shall *earn* his daily bread, has not been repealed. The struggle to-day for the necessities and comforts of life is as obligatory as ever, and with the rapid changes in our social life and increasing competition, we are forced more and more into narrower circles. The lads of this hour are men in the next, and within the hour they must become self-supporting. The law is inexorable.

The dental profession in this country is not recruited from the dilettanti of modern society—thank God—nor to any considerable extent from rich men's sons. The records of our colleges will not show ten per cent. of the students who are independent of their own earnings in obtaining their education, either already earned or their future mortgaged to return it. The grand achievements of the past and the hope and promise of a glorious future for our profession rest very largely upon this condition. Nothing makes success so valuable as the difficulties one overcomes in obtaining it.

Let me draw the picture from life. A young man with an academic education and limited means, with refined and artistic tastes, with natural abilities of that order that he is far more interested in the arts than in metaphysics or theology, is asking himself what occupation he shall adopt to obtain a living. As a boy, he could use his jack-knife with some skill, but if he attempted to swap jack-knives he was sure to get cheated. Commerce and trade are, therefore, not his sphere. His ambitions or his social surroundings prompt him to a more independent life than that of a mechanic with fixed hours and daily wages. Neither the practice of law nor the practice of medicine offers any field for the gratification of his tastes, but in an eminent degree the practice of dentistry does. And now comes *his* answer to the query which is agitating every dental society in the land. He says: "I have determined to be a dentist, and I am going to adopt that course of training which, with my limited means, will earliest make me master of my chosen profession. I do not wish to be a physician; I have no taste for nursing or gynecology any more than I have for law or theology. The general culture which a knowledge of those sciences gives I would like, but they do not concern me immediately. I can gratify any desire that I may have in that way after I am master of this one profession, providing that I do not find in that one full employment for scientific investigation. I find there are schools for dental students, and that graduates from those schools have become the most honored and skillful practitioners of dentistry that ever lived or ever will live, and with that encouragement before me, it is all I want."

Is the young man's reasoning wrong? He makes a practical application of conditions which Harvard, Yale and Cornell recognize, and all the advanced thought of the age endorses, viz., that a man's education should have, first, special reference to his chosen line of life, and that those branches of science which do not have a direct bearing upon that calling may be eliminated without harm.

At this point I may as well meet squarely the issue which is being forced upon us by some short-sighted enthusiasts, viz., that graduation in medicine is essential as a basis of dental education, and in the great to-morrow all dentists must be, first, graduates of medicine, and after that dentists. I can only liken these gentlemen to the passenger who sits on the rear platform of a railway train with his back toward the engine, and views the scenery only after it is passed.

The Vice-President of the Southern Dental Association, in his recent essay on dental education, would banish the dental degree, blot out dental colleges, and compel all students to obtain their dental education in medical schools, receiving the degree of Doctor of Medicine before allowed to practice. He has the hardihood to predict that such a condition will come about "within the next decade." But he is no wilder in his lunacy than the present President of the American Dental Association, who says: "Dentistry is not a profession, nor can it be except as it is medical, * * * and so sure as the sun shines the time will come when all dentists will be required to be medically educated." The trouble with both these eminent gentlemen is that they are sitting on the rear platform of the train, and have put the wrong end of the prophetical telescope to their eyes, and cannot discern that the inevitable progress of events is exactly in the opposite direction. Besides, their vision is blinded with a vague idea that M. D. tacked on to a dentist's name makes him, in the eyes of the community, and in fact, a better dentist; but no more foolish fallacy ever took possession of a misguided brain. The status of dentistry to-day shows it. The most skillful practitioners in the world, acknowledged so both by the community who seek their services and by their professional *confrères*, receive the reward of their merits utterly irrespective of graduation in medicine or the possession of that degree through favor.

But there are other gentlemen equally capable of casting the dental horoscope, who express themselves differently.

Before the American Dental Association in August, 1884, Dr. C. W. Spalding, whose eminence as an author, teacher and practitioner no one will question, made use of the following language:

"In this matter of preliminary medical education, it seems to me that the cart is put before the horse. Let us perfect ourselves in dentistry, and then, if we choose to adorn ourselves with a medical education, all very well. The difficulty is that we attempt to lay the foundation in the science which does not include our own at all, or, if at all, only to a small extent What is the difference between dentistry and medicine? The foundation principles upon which dentistry rests are anatomy, physiology, and chemistry, including also special pathology, therapeutics, and materia medica, with what we call operative and artificial dentistry. These compose the basis upon which the science of dentistry rests. It does not include an accurate knowledge of obstetrics and gynecology, nor an accurate knowledge of fevers and the like. Why should we educate ourselves, or require others to educate themselves, in branches that do not essentially belong to our profession? Why should we educate ourselves in non-essentials first, and in essentials afterward? Let us have the essentials first, and then, if other things can be added to advantage, that is a good thing."

Since writing the foregoing, I am in receipt of a letter from a practicing dentist, a graduate of medicine and a professor in a dental college, who speaks from experiment and experience. He says:

"My experience as one of the founders of an institution of learning for the purpose of educating medical graduates to practice dentistry has convinced me, against my will, that it is impossible to make skillful dentists, as a rule, from such material. A young man who would be a successful dentist must begin his study with his mind fixed, as far as possible, on his life-work. I am in favor of dentists studying medicine, but not engaging in medical study until after they become dentists. Oral surgery is a legitimate specialty of medicine; not so with dentistry. Oral surgery is taught in all schools of medicine, but who knows of a medical college in which men are taught dentistry? Dentistry, therefore, is not, nor can it ever be, a department of medicine, or a specialty of medicine in the sense that is ophthalmology, etc. This conclusion I have arrived at against my will. I have been forced to these convictions, and have abandoned a pet theory in consequence thereof, and these conclusions are not founded on theory, but facts which have been established on the firm ground of an expensive and hard-earned experience."

My own convictions must be already foreshadowed: dentists must be taught in dental schools, and dental schools must teach everything that a dentist needs to know which pertains to the practice of his profession. It is not a matter of any consequence whether such schools form a department in a university or maintain an independent organization, so long as the student secures the best training to fit him for his professional career. The question of a preliminary education is fast settling itself. The position which a dentist is to assume through life, as a professional man, demands at least what is known as a good academic education, and the dental colleges of to-day are recognizing this fact.

If I were to create a type which would be my ideal of a dentist, I would have him possessed of all the academical, classical and scientific knowledge that the world contains; and to crown it all, he should be a dentist. But such an idea is clearly chimerical. Life is too short and the capacity of the human mind too limited to make even an approach to it. Therefore, in the prescribed education of the dental student of to-day, only what is absolutely essential to make him master of his vocation is all that we have any right to require of him. After that, he may follow the bent of his natural tastes.

Let no man who may be disposed to criticise my opinions say that I am opposed to a thorough education, and more than all, I wish to disclaim any disrespect for, or any attempt to detract from, the value of a medical education. On the contrary, if any man feels that the study of and graduation in medicine is going to help him in the practice of dentistry, by all means encourage him. He cannot have too much knowledge, be it of medicine or any other science. But if he be a dentist and has obtained the degree of M. D. by graduation, let him not be vain enough to conceive that by its possession he has thus acquired skill superior to those who have not, and then flaunt his title in their faces with the Pharisaical air, "I am holier than thou."

I know many a dentist who, having obtained all the knowledge of his profession that the educational advantages of his times could give, and yet thirsting for a knowledge of cognate sciences, has, with the cares of a not very remunerative practice, nevertheless devoted all his spare hours, and eked from his scanty income the means to enable him to graduate in medicine. All honor to such men, I say. God bless them. The spirit which prompts them entitles them to a higher place than the acquirement of the degree ever gives them. But, while I am full of admiration for him who has earned his degree, I have equal contempt for the dentist who, without a medical education, succeeds in making himself so solid with the faculty of some medical college that they confer the degree upon him, and thereafter he plays the part of a sycophantic hanger-on to the outskirts of a profession which he could not by any possibility practice.

Another issue is being forced upon us and rapidly approaching a crisis. An International Medical Congress is announced to be held in this country, at Washington, in September, 1887, and dentists are asked to form a section of that congress. It would not require a very astute observer to divine, from my present discourse, what position on such a question I would be likely to take. As an independent profession, we have no business there. As dentists, we are out of place. A section of oral surgery is eminently proper, and if there are oral surgeons enough in the world who want a section all to themselves, by all means let them have it, but do not hitch dentistry on to the end of the tail of the medical kite to give it ballast for a higher flight. From more than one source have I heard this humiliating argument in its favor: "We ought to form this section because it will give us such an excellent opportunity to obtain recognition." Do those who talk about "recognition" in this connection realize what the word involves? In plain language it means that our condition heretofore has been one of inferiority and abasement, but by joining such a congress we shall immediately, by some sort of prestidigitation, be lifted into a very grand and inflential place. Dentistry in America needs no recognition that a medical congress can give. The only recognition which we need is that from all classes of the community, cultured and uncultured, and all professions, law, theology and medicine alike; a recognition that we are what we pretend to be—a benefit and a necessity to the health and comfort of the community. Any other recognition from a medical congress, even if filled with compliments, would be empty as sounding brass. If we are strong enough to form a section which will be a credit to dentistry in America, we are strong enough to have, in the not far-off future, a whole congress all to ourselves, and when that day arrives the eyes of the whole world will be centered upon us. We shall not be swamped in the multitude of specialties in medicine, like the poor relations who are invited to the feast but find themselves sitting at the second table. Fortunate it was for posterity that Chapin A. Harris and his colleagues were denied admission to the medical colleges. They builded wiser than they knew. Dentistry, independent, has grown with a vigor unparalleled. Grafted upon the medical stock and drawing its life, not from its own roots, but from the tainted juices of the parent tree, it would have been stunted and dwarfed beyond a possibility of redemption.

Dentistry has come to stay; not as a specialty, but as an honorable, dignified, learned, scientific, beneficent and independent profession. If to-day all the medical colleges, together with the entire medical profession, were blotted out, the practice of dentistry would not be injured in the least, nor would humanity, suffering from diseases of the teeth, be one whit the less cared for.

Dental colleges have come to stay. The degree of D. D. S. has come to stay, and dental societies—of which this New England Society is no mean type—have come to stay. Dentistry will exist long ages after you and I are forgotten. Even in that day of Paradise regained—when medicine will be no more, because disease has been banished from off the earth, and dental surgery has become a past history (because decay of the teeth has been prevented)—prosthetic dentistry then, alone of all the beneficent professions, will survive to supply the losses incident to advancing years, a blessing and a comfort to the toothless aged.

